# A Novel Variation in the Mitochondrial Complex I Assembly Factor NDUFAF5 Causes Isolated Bilateral Striatal Necrosis in Childhood

**DOI:** 10.3389/fneur.2021.675616

**Published:** 2021-06-10

**Authors:** Hongyan Bi, Hui Guo, Qianfei Wang, Xiao Zhang, Yaming Zhao, Jimei Li, Weiqin Zhao, Houzhen Tuo, Yongbo Zhang

**Affiliations:** ^1^Department of Neurology, Beijing Friendship Hospital, Capital Medical University, Beijing, China; ^2^Center for Medical Genetics and Hunan Key Laboratory of Medical Genetics, School of Life Sciences, Central South University, Changsha, China; ^3^CAS Key Laboratory of Genomic and Precision Medicine, Collaborative Innovation Center of Genetics and Development, Beijing Institute of Genomics, Chinese Academy of Sciences, Beijing, China

**Keywords:** bilateral striatal necrosis, NDUFAF5, mitochondrial complex I deficiency, whole-exome sequencing, novel variation

## Abstract

**Background:** Bilateral striatal necrosis (BSN) is characterized by symmetrical degeneration, predominantly of the caudate and putamen nucleus, in the basal ganglia. It is associated with numerous acquired and hereditary neuro-developmental and motor dysfunction-related pathological conditions. BSN results in high morbidity and mortality among infants and children, and its diagnosis is clinically challenging due to several overlapping disease phenotypes. Therefore, a precise genetic diagnosis is urgently needed for accurate genetic counseling and improved prognostic outcomes as well.

**Objective:** To identify novel missense mutations in the *NDUFAF5* gene as a cause of childhood BSN in members of a Chinese family and summarize the clinical characteristics of patients with the *NDUFAF5* gene mutations.

**Methods:** This study included a large family living in a remote northwestern area of China. Three siblings developed a neurological disorder characterized by generalized dystonia within the first decade of their lives. Cerebral computed tomography (CT) and magnetic resonance imaging (MRI) showed bilateral lesions of the putamen. Biochemical and genetic approaches were used to identify the cause of BSN.

**Results:** Sequence analysis showed no pathogenic variation in *PANK2, SLC25A19, SLC19A3*, and *NUP62* genes and in the entire mitochondrial genome as well. Whole-exome sequencing revealed compound heterozygous mutations consisting of *NDUFAF5*:c.425A > C(p.E142A) and c.836T > G (p.M279R). The father, a healthy sister, and a healthy brother of the affected siblings carried the c.836T > G mutation, and the mother carried the c.425A > C mutation. These variants were absent in 100 ethnically matched non-BSN controls. *In silico* analysis demonstrated that the E142A and M279R mutations in NDUFAF5 protein significantly perturbed the normal conformation of the protein due to alterations in the hydrogen bonding patterns around the evolutionarily conserved catalytic domains, leading to its loss of function in the early stage of mitochondrial complex I assembly.

**Conclusions:** We identified a novel compound heterozygous mutation (c.425A > C and c.836T > G) in the *NDUFAF5* gene as the potential cause of autosomal recessive childhood BSN, which extended the pathogenic variation spectrum of the *NDUFAF5* gene. This study provides substantial evidence for further improvement of genetic counseling and better clinical management of BSN affected individuals.

## Introduction

Bilateral striatal necrosis (BSN) represents a rare form of neurological disorders involving neostriata and is characterized by initial swelling of the dorsal striatum and neostriatum of the basal ganglia, especially the putamen and caudate nucleus, followed by progressive degeneration and necrosis of neuronal cells (1). It can be familial or sporadic in origin. BSN pathology may also involve globus pallidus, tegmental nucleus, and substantia nigra brain regions under various diseased conditions (2). The most commonly associated pyramidal symptoms include spasticity, hyperreflexia, and weakness primarily related to upper motor neuron degeneration, leading to developmental regression or cognitive as well as motor deficits (3).

Diverse pathogenic mutations in genes related to neuronal maturation, nuclear transport and mitochondrial respiratory system as well as severe biochemical defects have been implicated in various familial BSN pathogenesis, e.g., infantile autosomal recessive BSN-associated missense mutation (Q391P) in nucleoporin-62 (NUP62) (4); homozygous missense mutation c.373G > A in the *SLC25A19* gene encoding mitochondrial thiamine pyrophosphate transporter (5); *SLC25A19* gene mutation in pediatric recessive biotin-responsive basal ganglia disorder (6); homoplasmic mitochondrial 3697G > A mutation and DNA deletions (7); mitochondrial adenosine triphosphatase 6 (*ATP6*) gene mutations at 9176T > C and 8851T > C with symptoms resembling Leigh syndrome (8); childhood organic acidurias with parkinsonian rigidity (9); and Wilson's disease-like pathology visible in MRI as striatal necrosis in basal ganglia and atrophy (10), etc. Inherited forms of BSN manifest as dystonia, parkinsonian rigidity, eye movement abnormalities, chorea and athetosis overlap syndrome, myoclonus, seizures, and psychomotor retardation (11).

Biochemical and metabolic abnormalities in mitochondrial complex I have been frequently implicated in Leigh syndrome and BSN (12). Several pathogenic gene mutations have been identified in the components of complex I that are involved in ATP production and mitochondrial electrochemical gradient maintenance (13). Many of the NADH: Ubiquinone Oxidoreductase complex assembly factor (NDUFAF) family proteins act as the putative assembly factor for complex I, and their genetic mutations have been correlated with neurological disorders, including Leigh syndrome (14). Human *NDUFAF5 (C20orf7)* (MIM No.^*^612360), located in 20p12.1, encodes an important mitochondrial complex I assembly factor, arginine hydroxylase, which is imported from the nucleus into the mitochondrial inner membrane matrix. NDUFAF5 has been shown to hydroxylate NDUFS7 at Arg-73 at an early stage of mitochondrial complex I assembly (15). Biallelic pathogenic variants in *NDUFAF5* cause a rare type of autosomal recessive BSN, namely mitochondrial complex I deficiency, nuclear type 16 (MC1DN16, MIM No. #618238) (https://www.omim.org/entry/612360) and neonatal mitochondrial disease (16).

Here, we demonstrate a previously unknown compound mutation in *NDUFAF5* identified by whole-exome sequencing (WES) associates with a rare form of isolated BSN in childhood. Furthermore, with the help of protein structure prediction tools, we have investigated the implications of these missense mutations in functional modulations of NDUFAF5. Therefore, our findings highlight the importance of using an integrated approach that combines clinical evaluation with exome data analysis to reveal NDUFAF5 pathogenic mutation-associated disease mechanisms in lethal neuro-developmental diseases like BSN.

## Materials and Methods

### Subjects

This case study included 7 members of a family living in the northwest of China. The proband and two of her sisters were affected by BSN symptoms. This study was approved by the Ethics Committee of Beijing Friendship Hospital, Capital Medical University.

### Brain MRI and CT Scans

Brain MRI examination was conducted using a Discovery MR750 3.0T scanner. Conventional T1-weighted spin echo (T1WI) sequences were obtained with the following parameters: repetition time (TR) = 250 ms, echo time (TE) = 2.5 ms, FoV = 306 × 220 mm; parameters for conventional T2-weighted spin echo (T2WI) sequences: TR = 4,000 ms, TE = 157 ms, FoV = 306 × 220 mm. The slice thickness and the slice gap were 5 mm and 6.5 mm, respectively.

### Genetic Analysis

#### DNA Extraction

Blood samples were collected from all family members of the proband. Total DNA was extracted from the peripheral blood leukocytes using Blood and Tissue DNA isolation kit (Qiagen, Germany). The left biceps brachii muscle of the proband was biopsied, and the frozen sample was used for diagnostic studies.

#### Polymerase Chain Reaction (PCR) and Direct DNA Sequencing

Exonic and flanking intronic regions of nuclear genes *NUP62, SLC19A3, SLC25A19*, and *PANK2* and all mitochondrial genes including *MT-ATP6, MT-ND1, MT-ND3, MT-ND4*, and *MT-ND6* were amplified using specific primers (sequences available on request). Afterward, the coding regions and exon-intron junctions of these genes were analyzed by Sanger sequencing.

#### Whole-Exome Sequencing (WES) and Data Analysis

Exon-enrichment was carried out using SureSelect Human All Exon V5 Kit (Agilent Technologies, Santa Clara, CA, USA). The exon-enriched DNA libraries were subjected to paired-end sequencing with the HiSeq 2500 system (Illumina, San Diego, CA, USA). Raw data were filtered and processed using CASAVA v1.8 (Illumina). Calls with variant quality <20 were filtered out to obtain clean reads, and at least 95% of the targeted nucleotides were covered sufficiently to pass the thresholds for calling single-nucleotide mutations (SNPs) and small insertion-deletion (InDel) sequences. The sequencing reads were aligned to the human reference genome (Vhg19) using the Burrows-Wheeler Aligner (BWA), and PCR duplicates were removed using the Picard 1.27–1 software (http://picard.sourceforge.net/). A pipeline incorporating MuTect (Broad Institute) to identify the single-nucleotide variants and ANNOVAR (17) was employed for variant calling. FASTQ, BAM, and variant call format (VCF) files were then used for sequence analysis. The minor allele frequency (MAF) was analyzed using the 1000 Genomes Project database, SNP database (dbSNP), NHLBI exome sequencing project (ESP) (http://evs.gs.washington.edu/EVS/), and Exome Aggregation Consortium (ExAC) (http://exac.broadinstitute.org). Non-synonymous, loss-of-function, indel, duplication, and splicing variants were used to identify candidate variants. Protein biological function was predicted using the ConSurf (http://consurf.tau.ac.il) (18), Basic Local Alignment Search Tool (BLAST) (https://blast.ncbi.nlm.nih.gov/Blast.cgi), SIFT (http://sift.bii.a-star.edu.sg) and PolyPhen-2 (http://genetics.bwh.harvard.edu/pph2/) servers.

### Variant Confirmation by Sanger Sequencing

To confirm that the identified variants were responsible for BSN, we screened the 7 members in this family by Sanger sequencing using ABI 3500 system (Thermo Fisher, USA). Additionally, we analyzed 100 control specimens from our in-house sample library (obtained from individuals without BSN) to exclude polymorphisms.

### Molecular Modeling

Protein structure modeling was conducted using SWISS-MODEL with default parameters based on the available homologous models indexed in PDB database (https://swissmodel.expasy.org/). NDUFAF5 mutations were predicted using SWISS-MODEL (PDB: 2p35.1.A; Modeling GMQE score of 0.28, Modeling QMEAN score of 0.28–5.41; Seq Identity of 17.19%).

## Results

### Characteristics of the Participants

**Figure 1:**
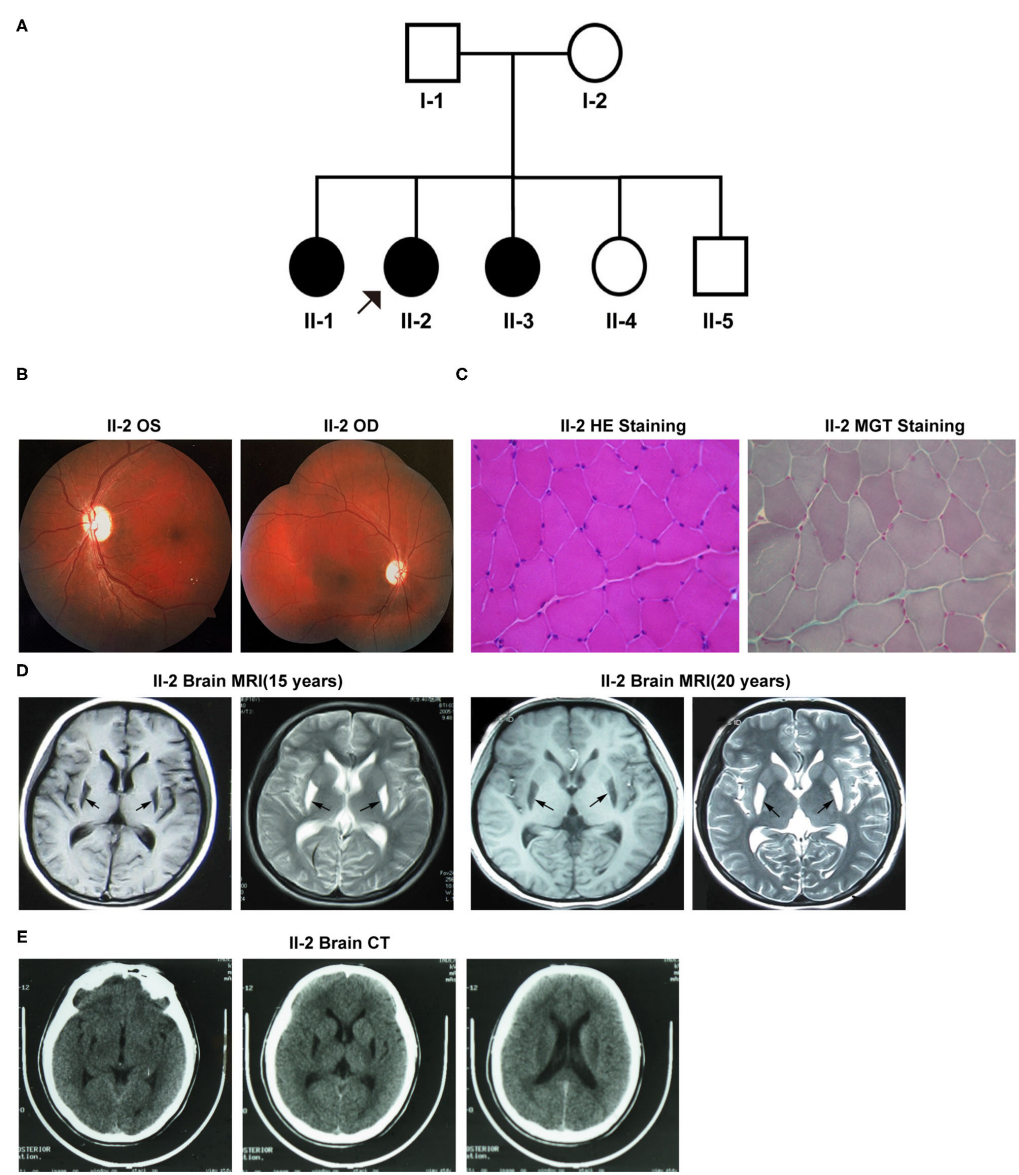
**(A)** The pedigree diagram of the family included in this study. **(B–E)** Clinical manifestations of the proband (II-2). **(B)** Fundus fluorescein angiography. **(C)** Muscle biopsy with specific staining. **(D)** Brain MRI at 15 and 20 years old. **(E)** Brain CT. OS, oculus sinister; OD, oculus dextrus; HE, hematoxylin-eosin; MGT, modified Gömöri trichrome.

Figure 1A exhibits the pedigree diagram of the participating subjects in this study as the proband (II-2), her father (I-1), mother (I-2), three sisters (II-1, II-3, and II-4), and one brother (II-5). BSN was diagnosed in the proband (II-2) at her early childhood and 2 of her sibling sisters (II-1 and II-3). The disease phenotypes included varying degrees of dystonic posture and movement disorders, dysarthric speech, dysphagia, and vision loss. The auditory and cognitive functions of the subjects were tested normal.

#### Patient II-2

Patient II-2 (the proband), the second child of her parents, was 20 years old at the first evaluation at our hospital. At the age of six, she started to exhibit the first onset of symptoms like gait difficulties, spasticity, and dysarthria aggravated by emotional stress at times, and the symptoms were progressively exacerbated with age. At her 13-years, she developed visual impairment due to optic nerve atrophy that lasted for more than 10 days. During her late adolescence (18 years of age), she exhibited the flexed dystonic postures that included inward rotation of legs and bilateral inversion of the foot, followed by dysphagia, dystonic movements, and decreased fine motor abilities. Neurological examinations revealed severe dystonic movements of the facial and trunk muscles, dysarthric speech, and other common pathological signs of BSN. A visual assessment revealed vision loss in both eyes-the visual acuity was 0.25D in the left eye and “counting fingers” in the right eye. Fundus fluorescein angiography showed bilateral pallor of the papilla in the retina (Figure 1B). Moreover, her muscle biopsy examination result was unremarkable for any pathological alterations in the structure of muscle cells (Figure 1C). However, her mitochondrial complex I NADH dehydrogenase activity was detected 86.8 nmol/min/mg mitochondrial protein (ref-range, ≥76.6 nmol/min/mg), which was much lower than that of her mother (129.5 nmol/l/min/mg mitochondrial protein). Brain MRI results at 15 and 20 years of age revealed abnormal signals with hypointense lesions on T1-weighted images and hyperintense lesions on T2-weighted images in the putamen of basal ganglia in the posterior region (Figure 1D). Both MRI and CT scans did not detect any signs of apparent calcifications in and around the affected brain regions (Figure 1E). Visual evoked potentials (VEPs) showed prolongation of the bilateral P100 latency, especially in the right eye, indicative of the demyelination of the optic nerve.

#### Patient II-1

**Figure 2:**
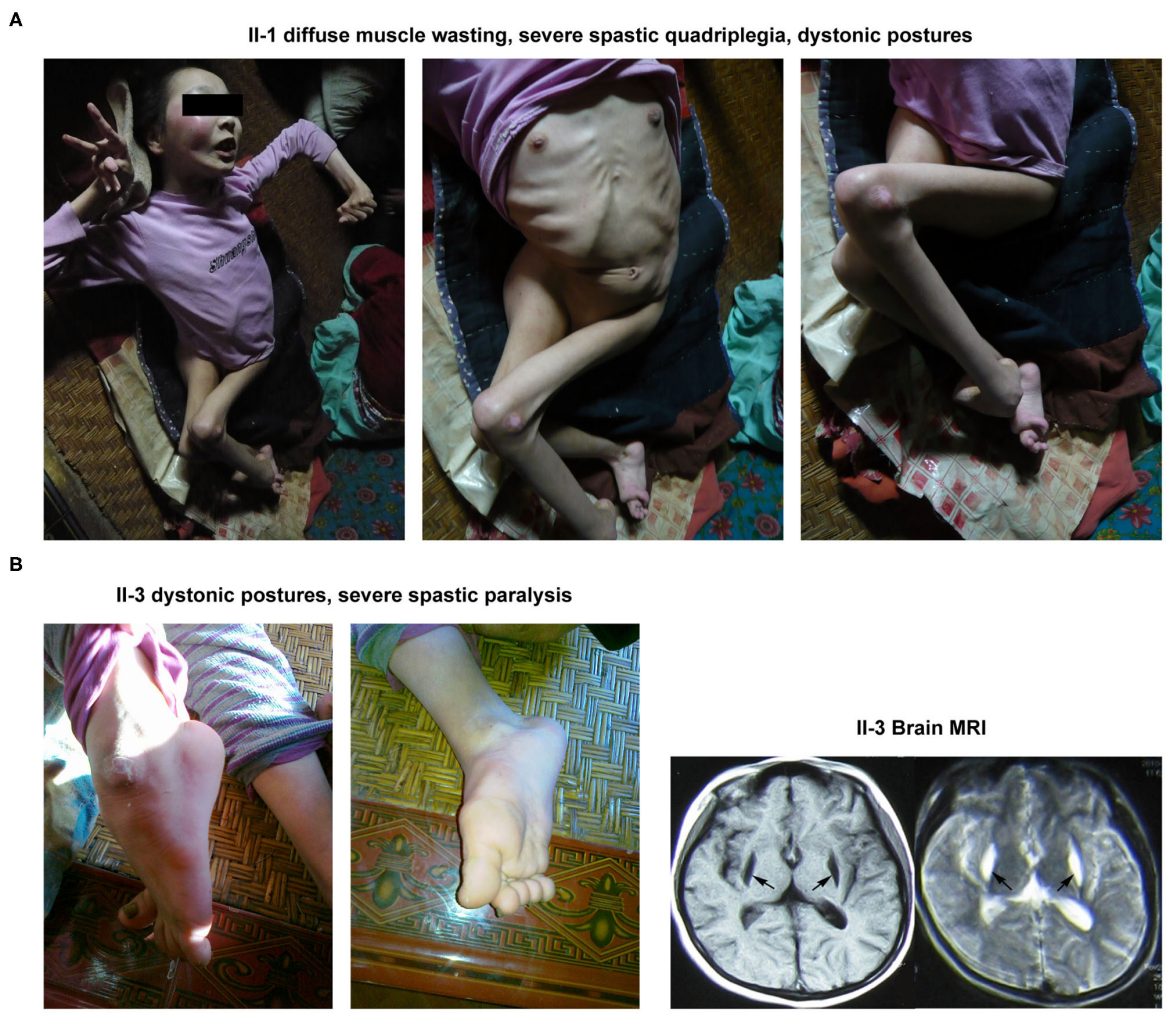
Clinical manifestations of the other 2 patients (II-1 and II-3) in this family. **(A)** The abnormal appearance of II-1. **(B)** The dystonic posture in lower limbs and brain MRI result of II-3.

The proband's elder sister was 23 years old at the time of her first diagnosis at the patient's home. She was first presented with walking impairment and dysarthric speech expression at 6 years of age. Progressive worsening of these pathological conditions led to the complete loss of autonomic gait and speech performances. Notably, she exhibited the most severe clinical symptoms of BSN amongst her family members. Neurological examinations revealed diffuse muscle atrophy leading to severe spastic quadriplegia and marked dystonic postures involving both the upper and lower limbs (Figure 2A).

#### Patient II-3

The younger sister of the proband was 18 years old during her first clinical examinations at her home. The patient had experienced 3 occasions of febrile convulsions between the age of 1 and 3. She began to display walking impairment and dysarthric speech expressions at the age of 5. At the time of clinical examinations, the patient was mostly bedridden and needed complete physical assistance with most of the daily motor performances, including walking. Neuromuscular examinations demonstrated dystonic postures involving both the upper and lower limbs as well as severe spastic paralysis of the right upper limb. Notably, her brain MRI examination revealed abnormal signals in the putamen (Figure 2B).

### Genetic Analysis

**Figure 3:**
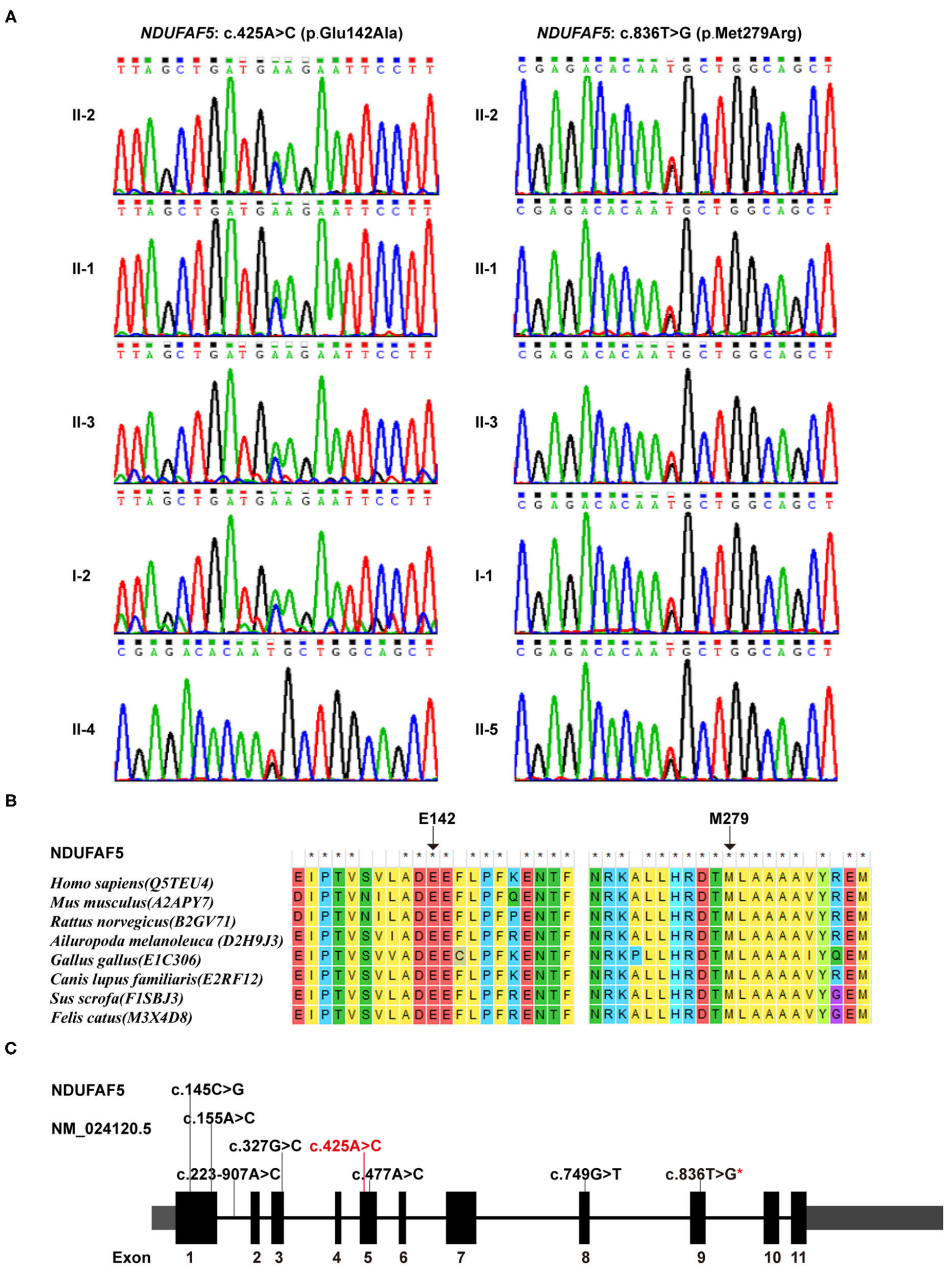
Genetic findings in this study. **(A)** Sequence variants and carrying status of each member in this family. **(B)** Conservatism of the 2 corresponding amino acids of the 2 missense variants among species. **(C)** The identified variants in *NDUFAF5* gene so far (Red characters represented the variant identified in this study; red star means the one identified both in this study and in other studies).

Our WES data provided a coverage depth of more than 99.60 × and an average mapping rate of 99.91%, which were sufficient to support our analysis, according to the previously published reports (19). Mutational analysis of these samples revealed a novel compound heterozygous variant in the *NDUFAF5* gene consisting of c. 425A > C (p.E142A) and c.836T > G (p.M279R) in 3 patients, suggesting the pathogenic role of *NDUFAF5* missense mutations in the childhood onset of BSN pathology. Furthermore, subjects I-1, II-4, and II-5 exhibited the presence of heterozygous *NDUFAF5* c.836T > G mutation, while subject I-2 carried the heterozygous c.425A > C mutation (Figure 3A). Furthermore, we matched this genomic sequence with 100 other unrelated healthy controls and concluded that these mutations were precisely related to BSN. Taken together, this study demonstrated that synergistic effects of two independent non-pathogenic paternal and maternal single-nucleotide mutations in the *NDUFAF5* gene could lead to an aggressive novel compound mutation initiating BSN pathogenesis at early childhood.

### Impact Prediction of the Variants

Individual impact prediction analysis of the heterozygous variants exhibited that the E142A variant had a disease risk score of 0.965 (sensitivity: 0.78, specificity: 0.95), and that of M279R variant was 0.930 (sensitivity: 0.81, specificity: 0.94). Additionally, analyses using ConSurf server and BLAST software demonstrated that the E142 and M279 residues were evolutionarily conserved among several mammalian species, including human, mouse, and rat (Figure 3B). Thus, the two mutational variants in the *NDUFAF5* gene led to two different amino acid substitutions in NDUFAF5 protein affecting the arginine hydroxylase enzymatic activities, leading to childhood BSN pathogenesis.

### Prediction of the Effects of the Mutations on Protein Conformation

**Figure 4:**
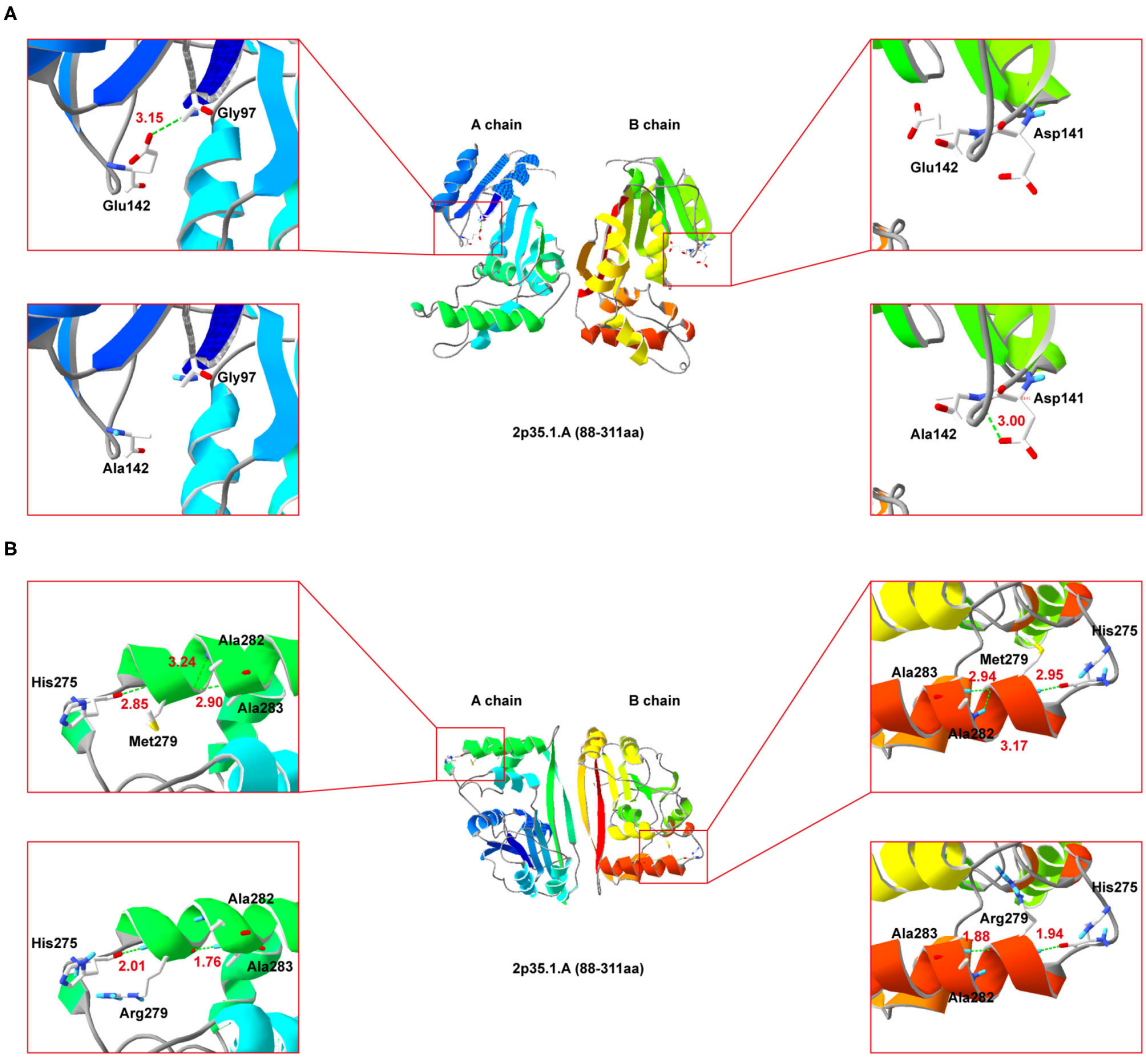
Secondary-structure of NDUFAF5 (residues 88–311) is shown. The intermediate region of **(A,B)** present a full picture of the structure of the protein dimer. Top left and top right of the **(A,B)** show magnified view of the outlined region containing the conserved residues. Lower left and lower right of **(A,B)** show magnified view of the outlined region containing the mutant residues.

Since NDUFAF5 forms homodimer, we performed simulated mutations on chain A (wildtype) and B (mutant), and then summed up the effects (Figure 4). Interestingly, E142A mutation significantly perturbed the normal hydrogen bond formation between E142 and G97 residues, leading to a conformational change in the mutant NDUFAF5. In chain A, the distance of the hydrogen bond between E142 and G97 was 3.15 Å, whereas it was absent in the B chain as A142 could not form hydrogen bonds with G97 (*top left and top right*; Figure 4A). Instead, a new hydrogen bond was formed in the B chain between A142 and D141 of 3.00 Å (*lower left and lower right;* Figure 4A). In variant M279R, a neutral methionine residue was replaced by a basic arginine moiety. Notably, M279R mutation significantly decreased the number of hydrogen bonds from 3 to 2 in the mutant NDUFAF5, resulting in decreased protein stability (M279, *top left and top right*, Figure 4B; Arg279: *lower left and lower right*, Figure 4B). Taken together, these results suggest that the novel missense mutation (E142A) could play a synergistic role in combination with a previously identified missense mutation (M279R) by negatively modulating the crucial enzymatic activities of NDUFAF5 under pathological conditions.

## Discussion

Here, we investigated a family with three siblings harboring compound heterozygous mutations in the *NDUFAF5* gene. The proband and her immediate elder and younger sisters were presented with the symptoms of childhood-onset generalized dystonia caused by BSN. The proband had vision loss due to optic nerve atrophy at her mid-childhood. Brain MRI examinations exhibited bilateral lesions in the putamen that were mainly confined to the posterior region that did not enlarge or regress over a 5-year period. The clinical course and radiological changes observed in these patients were inconsistent with those reported as the most common causes of BSN and related pathogenesis, such as infantile BSN (20), BSN and progressive polyneuropathy (21), biotin-responsive basal ganglia disease (22), Leber's hereditary optic neuropathy and dystonia (23), *MT-ATP6*-related neuropathy, ataxia and retinitis pigmentosa syndrome (24), Hallervorden–Spatz syndrome (25), and Huntington's disease (10). Using WES, we identified a previously unknown compound heterozygous mutation in *NDUFAF5* gene. Furthermore, protein conformational analysis of NDUFAF5 carrying missense mutations E142A and M279R revealed that considerable alterations in its conserved catalytic domains could be the possible reason for its inability to contribute to the normal physiological assembly of mitochondrial complex I subunits, leading to a severe loss of striatal neurons and motor impairments in BSN pathology.

NDUFAF5 protein possesses methyltransferase and arginine hydroxylase activity, which are critical for posttranslational modifications of other mitochondrial complex I assembly factors like NDUFS7 (26). This protein dysfunction has been implicated in mitochondrial complex I functional deficiency-induced fatal infantile neurodevelopmental diseases (27). Moreover, previous studies have shown the pathological relationship of various combinations of missense mutations in NDUFAF5 with familial BSN and related disease onset (28). These studies' main findings are summarized in Table 1, while the novel point-mutation c.425A > C (highlighted in red) is shown in Figure 3C. Notably, eleven of the thirteen patients described in these studies were not alive at the time of publications, with death occurring by the age of 7 years mostly due to cardiac arrest in 10 of the 11 patients, suggesting the aggressive influences of pathogenic *NDUFAF5* missense mutations on BSN pathogenesis and subsequent disease progression. RNAi-mediated knockdown of NDUFAF5 in control fibroblasts has exhibited decreased complex I activity, suggesting that the pathological complex I deficiency could have a direct mechanistic relationship with NDUFAF5 mutation-associated dysfunctions. Besides, alternative splicing of exon5 of the NDUFAF5 gene results in the production of shorter isoform under pathological conditions (29).

**Table 1:** Clinical features of patients with *NDUFAF5* variations reported in literature.

**References**	**Ethnicity**	Sex^*^	**Mutation**	**Onset age**	**Clinical features**	**MRI findings**	**Outcome**
Saada et al. (29)	Ashkenazi Jewish	M	c.749G > T, c.749G > T	12 m	Motor development retardation, ataxia, bilateral ptosis, optic atrophy, diffuse hypotonia	Symmetrical lesions of bilateral basal ganglia, striatum and cortical areas	Death at ~2.5 y
	Ashkenazi Jewish	M	c.749G > T, c.749G > T	12 m			Death at ~6 y
	Ashkenazi Jewish	F	c.749G > T, c.749G > T	12 m			Death at ~4.5 y
	Ashkenazi Jewish	F	c.749G > T, c.749G > T	12 m			Death at ~6 y
	Ashkenazi Jewish	F	c.749G > T, c.749G > T	12 m			Death at ~7 y
Fang et al. (30)	Chinese		c.212C > T, c.698G > T		Developmental delay and regression, seizures	Bilateral lesions of brainstem and basal ganglia	
Sugiana et al. (16)	Egyptian	M	c.719T > C, c.719T > C	Birth	Intrauterine growth retardation, facial dysmorphism, corpus callosum agenesis, ventricular septation, left diaphragmatic hernia, adrenal insufficiency	–	Death at ~7 d
Tong et al. (31)	Chinese	F	c.145C > G, c.836T > G	8 m	Neurodevelopmental delay, swallowing dysfunction, dyspnea	Bilateral medulla oblongata lesions	Death at 21 m
Gerards et al. (32)	Moroccan Moroccan	M M	c.477A > C, c.477A > C c.477A > C, c.477A > C	3 y 3 y	Dysarthria, dystonic posture, spastic quadriplegia, mental retardation	Caudate, putamen, substantia nigra and peri-aqueductal grey area lesions, bifrontal atrophy	Alive at 23 y Alive at 29 y
Simon et al. (20)	Taiwanese	F	c.155A > C, c.836T > G	6 m	Developmental delay, global hypotonia, difficulty swallowing	Symmetrical thalamic and midbrain lesions, corpus callosum dysgenesis	Death at 27 m
	Taiwanese	F	c.836T > G, c.836T > G	27 m	Vision loss, strabismus, nystagmus, muscle weakness, inability to walk	Hyperintense lesions in posterior fossa, caudate and cervical spinal cord	Death at 19 y
	Caucasian	M	c.327G > C, c.223–907A > C	3 m	Seizures, hypotonia, loss of vision, feeding difficulty	T2 hyperintensity in thalamus, midbrain, upper spinal cord	Death at 8 m
	Ashkenazi Jewish	M	c.327G > C, c.749G > T	5 m	Torticollis, nystagmus, swallowing and feeding difficulty	Bilateral lesions in thalamus, putamen and frontal lobes	Death at 17 m
This pedigree	Chinese	F	c.425A > C, c.836T > G	6 y	Generalized dystonia, spastic quadriplegia, dysphagia and dysarthria		Alive at 23 y
	Chinese	F	c.425A > C, c.836T > G	6 y	Generalized dystonia, optic atrophy, dysphagia and dysarthria	Abnormal symmetric signals in the posterior region of the bilateral putamen	Alive at 20 y
	Chinese	F	c.425A > C, c.836T > G	6 y	Generalized dystonia, febrile convulsions (1–3 y), dysphagia and dysarthria	Abnormal symmetric signals in the posterior region of the bilateral putamen	Alive at 18 y

* *M, Male; F, Female*.

Therefore, our results expand the genetic and phenotypic spectrum of BSN genetics to better understand the mechanistic implication of these missense mutations in different extents of disease aggressiveness. And the *NDUFAF5*-related BSN pathology should be included in the differential diagnosis of patients presenting with insidious dystonia in early childhood that progresses from gait disturbances to generalized dystonia with dysphagia and dysarthria.

## Data Availability Statement

The datasets generated for this study can be found in online repositories. The names of the repository/repositories and accession number(s) can be found below: SRA SRR14293161-SRR14293164 and BioSample SAMN18820150-SAMN18820153.

## Ethics Statement

The studies involving human participants were reviewed and approved by Ethics Committee of Beijing Friendship Hospital, Capital Medical University. Written informed consent to participate in this study was provided by the participants' legal guardian/next of kin. Written informed consent was obtained from the individual(s), and minor(s)' legal guardian/next of kin, for the publication of any potentially identifiable images or data included in this article.

## Author Contributions

HB designed and supervised the study and wrote the manuscript. HG and QW performed genetic testing and related data analyses. XZ, YZhao, JL, and WZ conducted the subject recruitment, initial medical examination, and iconography testing. HT and YZhan performed detailed examinations. All authors contributed to the article and approved the submitted version.

## Conflict of Interest

The authors declare that the research was conducted in the absence of any commercial or financial relationships that could be construed as a potential conflict of interest. The reviewer QW declared a shared affiliation, with no collaboration, with the authors HB, XZ, YZ, JL, WZ, HT, and YZ at the time of the review.
