# Corrigendum: A Novel Variation in the Mitochondrial Complex I Assembly Factor NDUFAF5 Causes Isolated Bilateral Striatal Necrosis in Childhood

**DOI:** 10.3389/fneur.2021.792230

**Published:** 2021-11-09

**Authors:** Hongyan Bi, Hui Guo, Qianfei Wang, Xiao Zhang, Yaming Zhao, Jimei Li, Weiqin Zhao, Houzhen Tuo, Yongbo Zhang

**Affiliations:** ^1^Department of Neurology, Beijing Friendship Hospital, Capital Medical University, Beijing, China; ^2^Center for Medical Genetics and Hunan Key Laboratory of Medical Genetics, School of Life Sciences, Central South University, Changsha, China; ^3^CAS Key Laboratory of Genomic and Precision Medicine, Collaborative Innovation Center of Genetics and Development, Beijing Institute of Genomics, Chinese Academy of Sciences (CAS), Beijing, China

**Keywords:** bilateral striatal necrosis, NDUFAF5, mitochondrial complex I deficiency, whole-exome sequencing, novel variation

**Error in Table**

On a recent occasion, we realized that in the original article, there was a mistake in Table 1 as published. **The citation numbers in the ***Table 1*** referring to the ***NDUFAF5*** mutations in various ethnic groups did not match the given reference order list in the published article. In Table 1, (1) reference 26 should be reference (****1****); (2) reference 27 should be reference 30; (3) reference 28 should be reference (****2****); (4) reference 12 should be reference 31; (5) reference 29 should be reference 32; and (6) reference 13 should be reference (****3****)**. The corrected *Table 1* appears below.

**Table 1:** Clinical features of patients with NDUFAF5 variations reported in literature.

**References**	**Ethnicity**	Sex^*^	**Mutation**	**Onset age**	**Clinical features**	**MRI findings**	**Outcome**
Saada et al. (1)	Ashkenazi Jewish	M	c.749G>T, c.749G>T	12 m	Motor development retardation, ataxia, bilateral ptosis, optic atrophy, diffuse hypotonia	Symmetrical lesions of bilateral basal ganglia, striatum and cortical areas	Death at ~2.5 y
	Ashkenazi Jewish	M	c.749G>T, c.749G>T	12 m			Death at ~6 y
	Ashkenazi Jewish	F	c.749G>T, c.749G>T	12 m			Death at ~4.5 y
	Ashkenazi Jewish	F	c.749G>T, c.749G>T	12 m			Death at ~6 y
	Ashkenazi Jewish	F	c.749G>T, c.749G>T	12 m			Death at ~7 y
Fang et al. (30)	Chinese		c.212C>T, c.698G>T		Developmental delay and regression, seizures	Bilateral lesions of brainstem and basal ganglia	
Sugiana et al. (2)	Egyptian	M	c.719T>C, c.719T>C	Birth	Intrauterine growth retardation, facial dysmorphism, corpus callosum agenesis, ventricular septation, left diaphragmatic hernia, adrenal insufficiency	–	Death at ~7 d
Tong et al. (31)	Chinese	F	c.145C>G, c.836T>G	8 m	Neurodevelopmental delay, swallowing dysfunction, dyspnea	Bilateral medulla oblongata lesions	Death at 21 m
Gerards et al. (32)	Moroccan	M	c.477A>C, c.477A>C	3 y	Dysarthria, dystonic posture, spastic quadriplegia, mental retardation	Caudate, putamen, substantia nigra and peri-aqueductal grey area lesions, bifrontal atrophy	Alive at 23 y
	Moroccan	M	c.477A>C, c.477A>C	3 y			Alive at 29 y
Simon et al. (3)	Taiwanese	F	c.155A>C, c.836T>G	6 m	Developmental delay, global hypotonia, difficulty swallowing	Symmetrical thalamic and midbrain lesions, corpus callosum dysgenesis	Death at 27 m
	Taiwanese	F	c.836T>G, c.836T>G	27 m	Vision loss, strabismus, nystagmus, muscle weakness, inability to walk	Hyperintense lesions in posterior fossa, caudate and cervical spinal cord	Death at 19 y
	Caucasian	M	c.327G>C, c.223–907A>C	3 m	Seizures, hypotonia, loss of vision, feeding difficulty	T2 hyperintensity in thalamus, midbrain, upper spinal cord	Death at 8 m
	Ashkenazi Jewish	M	c.327G>C, c.749G>T	5 m	Torticollis, nystagmus, swallowing and feeding difficulty	Bilateral lesions in thalamus, putamen and frontal lobes	Death at 17 m
This pedigree	Chinese	F	c.425A > C, c.836T > G	6y	Generalized dystonia, spastic quadriplegia, dysphagia and dysarthria		Alive at 23 y
	Chinese	F	c.425A > C, c.836T > G	6y	Generalized dystonia, optic atrophy, dysphagia and dysarthria	Abnormal symmetric signals in the posterior region of the bilateral putamen	Alive at 20 y
	Chinese	F	c.425A > C, c.836T > G	6y	Generalized dystonia, febrile convulsions (1-3 y), dysphagia and dysarthria	Abnormal symmetric signals in the posterior region of the bilateral putamen	Alive at 18 y

* *M, Male; F, Female*.

**Missing Citation**

In the original article **References 30, 31, and 32** were not cited/included in the published article. The citation has now been inserted in Table 1, under the section ***Discussion***.

**New References to be Added in the continuing order:**

30. Fang F, Shen Y, Shen DM, Liu ZM, Ding CH, Zhang WC, et al. [Clinical and genetic characteristics of children with Leigh syndrome]. *Zhonghua er ke za zhi* = *Chinese J Pediatr*. (2017) 55:205–9. doi: 10.3760/cma.j.issn.0578-1310.2017.03.008
31. Tong W, Wang Y, Lu Y, Ye T, Song C, Xu Y, et al. Whole-exome sequencing helps the diagnosis and treatment in children with neurodevelopmental delay accompanied unexplained dyspnea. *Sci Rep*. (2018) 8:5214.
32. Gerards M, Sluiter W, van den Bosch BJ, de Wit LE, Calis CM, Frentzen M, et al. Defective complex I assembly due to C20orf7 mutations as a new cause of Leigh syndrome. *J Med Genet*. (2010) 47:507–12. doi: 10.1136/jmg.2009.067553

The authors apologize for this error and confirm that it does not change the scientific conclusions of the article in any way. The original article has been updated.

## Publisher's Note

All claims expressed in this article are solely those of the authors and do not necessarily represent those of their affiliated organizations, or those of the publisher, the editors and the reviewers. Any product that may be evaluated in this article, or claim that may be made by its manufacturer, is not guaranteed or endorsed by the publisher.
